# The Epidemiology and Clinical Manifestations of Dysexecutive Syndrome in Parkinson’s Disease

**DOI:** 10.3389/fneur.2012.00159

**Published:** 2012-11-08

**Authors:** Roberto Ceravolo, Cristina Pagni, Gloria Tognoni, Ubaldo Bonuccelli

**Affiliations:** ^1^Department of Neuroscience, University of PisaPisa, Italy

**Keywords:** Parkinson’s disease, dysexecutive syndrome, executive function, neuropsychological test, cognitive impairment

## Abstract

This mini-review summarizes the evidence of the cognitive and behavioral features of dysexecutive syndrome in Parkinson’s disease (PD). Deficits in response inhibition, set-shifting, mental flexibility, and strategy have been frequently described from the earliest stages of PD, although there are inconsistencies in study findings due to the complexity of the executive function (EF) construct and methodological limitations. Behavioral disorders of PD, e.g., apathy, distractibility, perseverative behavior, and impulse-control disorders, may be viewed as the other side of dysexecutive syndrome. Despite the interrelationship between the cognitive and behavioral domains, some reports reveal that the two syndromes may be dissociated, suggesting that both aspects must be clinically assessed. EFs are widely associated with the prefrontal areas, although dysexecutive syndrome may be observed in patients with damage to other brain regions. EFs drive numerous abilities essential to daily life, such as prospective remembering and language comprehension, which may be impaired in PD subjects. Considering the impact of dysexecutive syndrome on independence and quality of life, early detection of executive impairment is crucial in the management of PD.

## Introduction

A growing body of recent studies on cognition in Parkinson’s disease (PD) have indicated that different cognitive domains may be affected from the early stages of PD (Dujardin et al., 2004; Foltynie et al., 2004; Muslimovic et al., 2005; Aarsland et al., 2009a; Elgh et al., 2009; Martin et al., 2009). These include memory, language, attention, visuospatial and visuoconstructive abilities, and executive functions (EFs). Several studies have assessed the cognitive deficits in PD by using Petersen’s definition of mild cognitive impairment (MCI; Petersen, 2004). The Movement Disorder Society’s commissioned task force highlighted that MCI is common in non-demented PD subjects, with a mean prevalence of 27% (range: 19–38%). PD-MCI is associated with increasing age, disease duration, and disease severity (Litvan et al., 2011), and may be considered to be a precursor to dementia (Aarsland et al., 1996, 2003; Hely et al., 2008).

In this regard, executive dysfunction appears to be the most pronounced impairment (Dubois and Pillon, 1997; Zgaljardic et al., 2003), although results differ depending upon the tests used and the heterogeneity of patient groups. Disorders of EF may progress to dysexecutive syndrome, a poorly defined nosological entity, which may be caused by various diseases. According to diagnostic criteria proposed by Godefroy et al. (2010), dysexecutive syndrome includes cognitive (e.g., deficits in response inhibition, set-shifting, and information generation) and/or behavioral (e.g., apathy, distractibility, perseverative behavior) alterations. In their validation study, Godefroy et al. (2010) reported a frequency of cognitive and behavioral dysexecutive syndromes of 39 and 42%, respectively, in PD patients.

Some evidence highlights that executive dysfunction in non-demented PD patients predisposes to an increased impairment of daily activities (Kamei et al., 2008). Because of the impact on the patient’s independence and quality of life, early detection of executive impairments is crucial in the management of PD.

## Cognitive and Behavioral Aspects of Executive Dysfunction in PD

“Executive function” is an umbrella term that includes different aspects of goal-directed behavior (Jurado and Rosselli, 2007). In this construct, abilities such as planning, problem-solving, top-down mechanisms of attention control, and working memory are included. There is an ongoing debate on the unitary (Duncan and Miller, 2002) versus non-unitary character of EF (Stuss and Benson, 1986; Shallice, 2002), and no gold standard neuropsychological tools have been agreed upon (Royall et al., 2002).

Although the EF construct is complex, classical “frontal” tests have been widely used in clinical practice and research on cognition in PD. These tests explore EF as a whole (e.g., Frontal Assessment Battery, Behavioral Assessment of Dysexecutive Syndrome) or some aspects of it, such as divided attention (e.g., Trail Making Test B), response inhibition (e.g., Stroop Interference Test), working memory (e.g., Digit Span forward and backward, Dual-Task paradigms), mental flexibility (e.g., Wisconsin Card Sorting Test, Verbal Fluency Tasks), and planning (e.g., Tower of London). For an explanation of these tests, see Lezak et al. (2004). Several studies have reported deficits on these tasks in early stage PD patients (Fama et al., 2000; Grace et al., 2005; Janvin et al., 2006; de Frias et al., 2007; Lima et al., 2008; Muslimovic et al., 2008; Siegert et al., 2008; Aarsland et al., 2009a), but the exact pattern of impairment is still under debate (Muslimovic et al., 2007; see for a review Watson and Leverenz, 2010). This impairment is often evident in time-constraint tests, suggesting a psychomotor slowing in PD subjects (Owen et al., 1992; Robbins et al., 1994; Kamei et al., 2008). Furthermore, some reports indicate a relative sparing of performance in EFs tasks in PD subjects (Auriacombe et al., 1993; Farina et al., 2000; Cools et al., 2001; Colman et al., 2009).

Kudlicka et al. (2011) conducted a meta-analysis of the literature focusing on executive impairment in PD. In particular, they searched for studies of PD patients without dementia or depression and a Hoehn and Yahr score of I–III. Significant difficulties were found in PD subjects compared with controls across all measures of EF (i.e., verbal fluency [phonemic, semantic, and alternating], digit span backward, Wisconsin Card Sorting Test, Stroop test, Trail Making Test B). Hence, controlling for the influence of disease characteristics, it is possible to reduce the inconsistency in study findings. Nevertheless, authors highlighted many methodological limitations in EF studies; in particular, the general absence of a background formal model of EFs that specifies the various subcomponents and their assessment methods.

Classical theoretical views of EFs include the multicomponent model of Working Memory (Baddeley and Hitch, 1974, 1994) and the Supervisory Attentional System framework (Norman and Shallice, 1986). The former distinguishes four components: the *central executive*, which controls attention and manipulation of information, the *phonological loop*, and the *visual sketchpad*, two subcomponents involved in short-term storage of verbal and visuospatial information, and the *episodic buffer*. The latter postulated a *supervisory system* that plays a role when faced with novel situations, while the *contention scheduling* automatically responds to routine situations.

The debate regarding the role of the central executive and the supervisory attentional system has encouraged studies that better clarify the concept of EFs. Nevertheless, some authors do not support the concept of an undifferentiated central executive or supervisory system and propose a flexible assembly of attentive control processes (Stuss and Alexander, 2007).

Executive deficits along with visuospatial and memory and language impairments could also predict dementia in cohort studies (Jacobs et al., 1995; Mahieux et al., 1998; Levy et al., 2002; Hobson and Meara, 2004; Janvin et al., 2005). Dysexecutive syndrome has been described as a key feature of PD dementia, supporting the assumption that executive deficits might evolve into dementia. Conversely, Williams-Gray et al. (2007), in a longitudinal study of an incident population-based cohort of PD patients, found that the posterior cortically based deficits, rather than executive impairments, appeared to evolve into dementia over a 3.5-year follow-up.

It is important to consider that neuropsychiatric symptoms of PD may be associated with cognitive impairment. Apathy, depression, and anxiety are present in one-third of PD patients (Aarsland et al., 2009b). Major depression in PD has been associated with deficits in some cognitive domains, specifically memory, and EFs (Kuzis et al., 1997; Norman et al., 2002; Costa et al., 2006), presumably as a consequence of difficulty in mobilizing cognitive resources to complete tasks (Hasher and Zacks, 1979; Zakzanis et al., 1998). Varanese et al. (2011) reported that apathy, but not depression, was associated with specific disorders of recall and EFs in PD subjects. The authors highlighted the specific impairment in generating new, efficient cognitive strategies, according to the model of “cognitive inertia” (Levy and Dubois, 2006). Deficit of episodic memory in PD may thus be viewed as an epiphenomenon of poor strategy implementation in encoding and retrieval phases (Brønnick et al., 2011), rather than a primary disorder. They propose apathy to be an early manifestation of dysexecutive syndrome in PD (Varanese et al., 2011), according to Godefroy’s criteria. Weintraub et al. (2005) pointed out that planning deficits and diminished inhibitory control, two dimensions of executive impairment in PD, were associated with decreased motivation and increased motor slowing, respectively. Decreased psychomotor speed (bradyphrenia) and fatigue may be taken into account for their potential effect on test performance.

Although the emerging picture shows an interrelationship between cognitive and behavioral features, some reports reveal that the two syndromes may be dissociated (Eslinger and Damasio, 1985; Godefroy, 2003). This suggests that both behavioral and cognitive domains must be clinically assessed. Besides clinical judgment, the assessment of behavioral disorders is made through structured interviews using, for example, the Neuropsychiatric Inventory (Cummings et al., 1994), the Cambridge Behavioral Inventory (Wedderburn et al., 2008), the Frontal Behavioral Inventory (Kertesz et al., 1997). As previously mentioned, apathy is a frequent behavioral change in the early stages of PD, but distractibility and perseverative behavior have also been reported (Sharpe, 1990; Ebersbach et al., 1994; MacHado et al., 2009). Recently, impulse-control disorders (ICDs), which PD patients may develop during dopamine (DA) replacement therapy, are being increasingly recognized in PD patients as indicative of dysexecutive syndrome.

Impulse-control disorders include different behaviors such as pathological gambling, hypersexuality, compulsive eating, compulsive hobbyism, and compulsive medication use. Studies reported an overall prevalence of 5.9–6.6%. (Weintraub et al., 2010; Voon et al., 2011). ICDs have been associated with cognitive dysfunction. Findings suggested that PD patients with ICDs are impaired in frontal tasks (e.g., spatial planning and set-shifting), and specific neuropsychological correlates have been reported (Vitale et al., 2011). Conversely, other authors failed to find a dysexecutive basis for pathological gambling (Siri et al., 2010). Nevertheless, ICDs are also associated with personality traits (Farnikova et al., 2012), genetic susceptibility (Lee et al., 2009), and neuropsychiatric symptoms (Voon et al., 2011).

## Neurobiological Basis of Executive Deficits in Non-Demented PD Subjects

A number of relevant studies have demonstrated that EFs are widely associated with the prefrontal areas. Specifically, the dorsolateral prefrontal cortex is involved in working memory (Petrides, 2000) and cognitive flexibility (Milner, 1963; Goldman-Rakic, 1987), while the ventrolateral and orbital prefrontal cortex are involved in decision-making processes and acquisition and reversal of stimulus–reward association (Nauta, 1971; Rolls, 2000). Conversely, neuropsychological studies show that mediofrontal lesions are the basis of behavioral impairment (Eslinger and Damasio, 1985; Bechara et al., 1998). However, dysexecutive syndrome may be also observed in patients with damage to other brain regions (Morris et al., 1990; Owen, 2004).

Cognitive disabilities in PD have been associated with DA depletion in the prefrontal cortex and caudate nucleus (Roberts et al., 1994; Lewis et al., 2003; Carbon et al., 2004; Grahn et al., 2008). In this regard, the concept of corticostriatal loops (Alexander et al., 1986; Cummings, 1993; Lichter and Cummings, 2001) is a central model that highlights the functional connections between the frontal cortex and the striatum.

The spatiotemporal progression of the DA depletion moves from the putamen and the dorsal caudate nucleus and only later progresses to the more ventral parts of the striatum and to the mesocorticolimbic dopaminergic system (Leh et al., 2010). Within the caudate nucleus, DA depletion was greatest in the rostrodorsal extent of the head of the nucleus, an area connected to the dorsolateral regions of the frontal lobe (Yeterian and Pandya, 1991). Conversely, the ventral region of the caudate, connected to more ventral regions of the frontal lobe, is relatively spared in early PD.

These findings are consistent with the model of frontostriatal cognitive degeneration in PD proposed by Owen et al. (1998); see for a review Owen (2004). As these authors suggested, higher-level EFs, such as manipulation, strategies, and planning, that rely on the integrity of the dorsolateral frontal cortex, may be more susceptible than basic mnemonic functions, such as encoding and retrieval, which are assumed to depend on more ventral frontal regions (Petrides, 1994; Owen, 2000). This uneven dopaminergic loss has implications for medication effects on cognition. In fact, anti-parkinsonian drugs may differentially modulate cognitive performance, depending on the neural substrate underlying the cognitive function being tested (Gotham et al., 1988). According to the “l-DOPA overdose hypothesis in PD” (Cools et al., 2001), dopaminergic therapy improves the dorsal-caudate-related tasks, by normalizing DA levels in this severely depleted area, while it worsens the ventral-striatum-related tasks. This paradoxical effect is due to the “overdosing” of a relatively normal ventral striatum (Cools, 2006; Cools et al., 2007). This phenomenon is particularly evident in ICDs, as mentioned above.

The pattern of cognitive impairment in PD evolves according to progression of DA depletion within the striatum, which parallels the motor deficits of the disease (Owen et al., 1992, 1993).

However, on the basis of neuroimaging studies, some authors hypothesized that not only the nigrostriatal, but also the mesocortical dopaminergic substrate may play a significant role in cognitive dysfunction in PD (Monchi et al., 2007). Moreover, although a dopaminergic substrate of executive dysfunction is well recognized, other neurotransmitter systems (e.g., noradrenergic, serotoninergic, and cholinergic systems) may contribute to the cognitive dysfunction observed in PD (Grahn et al., 2008). Similarly, cortical Lewy body pathology, which can occur even in the early stages, may contribute to the development of dementia (de la Fuente-Fernández and Calne, 1996; Braak et al., 2003).

## Executive Functions in Daily Life

Executive functions direct the sequencing and execution of complex goal-directed activities (e.g., cooking, dressing, check writing, and housework) so that EF impairment has emerged as a robust determinant of functional status and disability (Royall et al., 1998, 2000).

Executive functions drive other abilities essential to daily life, such as prospective remembering. Prospective memory (ProM) is a multifaceted function, concerned with the ability to implement intended actions in the future (Kliegel et al., 2011). Successful prospective remembering is crucial in all daily contexts (e.g., remembering to take medication). ProM is dependent on two components: a retrospective component supporting the retrieval of the intention content from long-term memory; and a prospective component involving real-time detection or recognition of prospective cues. The latter component requires a series of processes, including planning, maintaining multiple goals in working memory, and shifting attention, which are the subcomponents of EFs that are impaired early in PD. Previous findings from dual-task paradigms, which are based on ProM paradigms, showed that performance in the primary task increasingly worsened with increasing cognitive load of the secondary task (Brown and Marsden, 1991; Wu and Hallett, 2008; Ma et al., 2009; Proud and Morris, 2010).

Many investigations of ProM in PD found impairments in the prospective component of both event based tasks (i.e., the action is cued by an event; Katai et al., 2003; Kliegel et al., 2005; Whittington et al., 2006) and time-based tasks (i.e., the action is cued by time; Costa et al., 2008a; Raskin et al., 2011). A subtle ProM dysfunction has been described in individuals with untreated, newly diagnosed PD (Pagni et al., 2011). ProM deficits in PD are also related to task difficulty (Altgassen et al., 2007) and absence of test cueing (Foster et al., 2009). Kliegel et al. (2011) proposed that the processes specifically impaired in PD subjects are intention formation and intention initiation, which rely on planning and monitoring abilities. Moreover, there is some evidence that ProM performance in PD is associated with a low score on working memory and executive tests. These findings indicate that ProM deficits seen in PD could be due to executive impairment. Considering that ProM deficits are frequently associated with a reduced quality of life in neurological patients (Fish et al., 2010), it will be of interest to explore these aspects from the beginning of the disease.

Another interesting viewpoint of EFs in daily life is language. Sentence comprehension difficulties have been described in PD subjects. These comprehension deficits are particularly evident with syntactically complex passive sentences (i.e., sentences in which the thematic roles are not in the canonical positions, such as in passive sentences; Lieberman et al., 1992) and longer sentences (Caplan and Waters, 1999; Skeel et al., 2001; Grossman et al., 2003). Several aspects of executive functioning, such as inhibition, set-shifting, sequencing, and information-processing speed have been associated with language comprehension in PD (Hochstadt et al., 2006; Hochstadt, 2009). These results have theoretical implications regarding the functional basis of language impairment. Current views hold that there does not appear to be a language faculty totally modular and independent of EF influence.

As for treatment effect, Grossman et al. (2001) found that PD patients’ performance on sentence comprehension worsened while they were off therapy, according to the overdose hypothesis. Similarly, PD subjects were selectively impaired in intention autonomous retrieval of a time-based ProM task when they were in the “off-state” (Costa et al., 2008b).

## Impact on Quality of Life

There is a lack of knowledge about the subjective experience of executive dysfunction and the effect on quality of life. Awareness of cognitive impairment is crucial in order to provide appropriate support for patients and caregivers. Moreover, anosognosia (the lack of awareness of cognitive dysfunction) is a supportive deficit of behavioral dysexecutive syndrome (Godefroy et al., 2010).

Koerts et al. (2012) found that patients with mild-to-moderate PD were aware of their executive problems in daily life, and reported consistently more problems with respect to healthy controls. Nevertheless, authors failed to find an association between executive complaints and performance on tests measuring EFs. This is probably due to the lack of ecological validity of the classical executive tests, although other sources of bias in subjective reports have been described (e.g., the influence of mood, see Marino et al., 2009).

Some attempts have been made to reproduce the daily life situations in which EFs are required, which are generally unstructured regarding goals, strategies, and possible solutions. Koerts et al. (2011) have developed an instrument, the cognitive effort test (CET), that resembles multitasking in daily life. Controls and PD patients showed different strategic approaches on CET to parallel task execution versus sequential task execution. In this sense, PD patients, especially those with moderate PD, seem to take their psychomotor speed retardation into consideration when planning and executing tasks.

From a rehabilitation perspective, PD patients may take advantage of cognitive training (Sinforiani et al., 2004; Brauer and Morris, 2010; París et al., 2011; Calleo et al., 2012), although very few studies are available. Interventions, such as goal-management training (Levine et al., 2000), in which subjects were trained to explore the most useful strategies to comply with various tasks, could be successfully applied. In this regard, Costa et al. (2012) argued that interventions focusing on shifting abilities (e.g., dual-tasks and switching tasks) may be useful, considering that these abilities are a subprocesses of other important functions (such as ProM and language comprehension abilities) in early impaired PD subjects.

## Conclusion

In summary, the general picture emerging from the available literature reveals that dysexecutive syndrome in PD may have different cognitive and behavioral features. Formal test-based assessments might be able to evaluate the different executive processes. Questionnaires are useful for the assessment and follow-up of behavioral disorders. A correct assessment of both the cognitive and behavioral domains is also crucial to selecting the best treatment options.

## Conflict of Interest Statement

The authors declare that the research was conducted in the absence of any commercial or financial relationships that could be construed as a potential conflict of interest.
